# Genome-wide identification and expression analysis of *ClLAX*, *ClPIN* and *ClABCB* genes families in *Citrullus lanatus* under various abiotic stresses and grafting

**DOI:** 10.1186/s12863-017-0500-z

**Published:** 2017-04-07

**Authors:** Chenliang Yu, Wenqi Dong, Yihua Zhan, Zong-an Huang, Zhimiao Li, Il Seop Kim, Chenghao Zhang

**Affiliations:** 1 Vegetable Research Institute, Zhejiang Academy of Agricultural Sciences, Hangzhou, 310021 China; 2 State Key Laboratory of Plant Physiology and Biochemistry, College of Life Sciences, Zhejiang University, Hangzhou, 310058 China; 3 Institute of Vegetable Sciences, Wenzhou Academy of Agricultural Sciences, Wenzhou Vocational College of Science and Technology, Key Lab of Crop breeding in South Zhejiang, Wenzhou, 325014 China; 4 Dempartment of Horticulture, Kangwon National University, Chuncheon, 200-2071 Korea

**Keywords:** *ABCB*, Abiotic stresses, Grafting, *LAX*, *PIN*, Watermelon

## Abstract

**Background:**

Auxin plays an important role in regulating plant growth and development as well as in the response of plants to abiotic stresses. Auxin is transported by three kinds of major protein families, including the AUXIN RESISTANT 1/LIKE AUX1 (AUX⁄LAX) influx carriers, the PIN-FORMED (PIN) efflux carriers and the ATP binding cassette B/P-glycoprotein/Multidrug-resistance (ABCB/MDR/PGP) efflux/condition carriers. The biological function of several auxin transporter genes has been well characterized in *Arabidopsis thaliana*. However, their function in response to exogenous auxin and abiotic stresses in watermelon (*Citrullus lanatus*. L) remained unknown.

**Results:**

Here, the latest updated watermelon genome was used to characterise the *ClLAX*, *ClPIN* and *ClABCB* family genes from watermelon. The genome-wide analysis of the *ClLAX*, *ClPIN* and *ClABCB* family genes, including chromosome localisation, gene structure, and phylogenic relationships, was carried out. Seven *ClLAXs*, 11 *ClPINs* and 15 *ClABCBs* were mapped on 10 watermelon chromosomes. The expression profiles of the *ClLAX*, *ClPIN* and *ClABCB* genes under exogenous indole-3-acetic acid and various abiotic stresses (salt, drought, and cold stresses) treatments were performed by quantitative real-time PCR (qRT-PCR). The transcriptional level of majority *ClLAX*, *ClPIN* and *ClABCB* genes were changed by abiotic stresses in both shoots and roots. We also analysed the expression levels of *ClLAX*, *ClPIN* and *ClABCB* genes in graft response.

**Conclusion:**

Analysis of the expression patterns of *ClLAX*, *ClPIN* and *ClABCB* genes under salt, drought, cold treatment and grafting response helps us to understand the possible roles of auxin transporter genes in watermelon adaptation to environmental stresses.

**Electronic supplementary material:**

The online version of this article (doi:10.1186/s12863-017-0500-z) contains supplementary material, which is available to authorized users.

## Background

Auxin is a very important plant hormone involved in regulating many processes of plant growth and development, such as root formation, apical dominance, inflorescence and phyllotaxy development, vascular tissue differentiation, fruit maturation and responses to illumination and gravity. Abnormal phenotypes are observed in plants, which are caused by excessive or insufficient concentrations of endogenous auxin [1]. Plants are inevitably subject to abiotic stresses such as salinity, cold, high temperature and drought during the life cycle. Auxin plays a key role in plant response to stress [2, 3] and environmental stress response relies on auxin homeostasis within different plant tissues [4]. The homeostasis of auxin is often disturbed by abiotic stress, which leads to the change of plant growth and development [5, 6].

Auxin is primarily synthesised in apical meristems and developing leaf tips, then transported to distal target tissues either through the bulk flow in stem vascular tissues in a non-polar free diffusion or actively in a polar transport [7]. Auxin transport exhibits polarity, which is unique among all phytohormones. The polar transport of auxin is mediated through the auxin carriers, including AUXIN RESISTENT1/LIKE AUX1 (AUX/LAX) influx carrier, PIN-FORMED (PIN) efflux carriers, and ATP binding cassette B/P- glycoprotein/Multidrug-resistance (ABCB/MDR/PGP) efflux/condition carriers [8–10].

*AUX*/*LAX* family is a subclass of amino acid superfamily recognized as auxin input carrier family. *AtAUX1* is the first *AUX/LAX* family gene cloned in *Arabidopsis*, which encoded a protein containing 11 transmembrane structure [8]. Mutations of *AUX/LAX* show the auxin-related developmental defects in *Arabidopsis thaliana. Ataux1* mutants are agravitropic and selective resistant to auxin [11]. They are insensitive to indole-3-acetic acid (IAA) and (2, 4-dichlorophenoxy)-aceticacid (2, 4-D). Only free diffusion of naphthalene-1-acetic acid (NAA) can restore the gravitropism of *ataux1* [11, 12]. *AtLAX3* and *AtAUX1* co-ordinately regulate lateral root development by regulating the emergence and initiation of lateral root primordia [13, 14]. AtAUX1 and AtLAX3 are high-affinity auxin transporters by auxin uptake experiments in heterologous expression systems [13, 15, 16]. Disruption of the *AtLAX2* gene results in increasing division of the cells in the quiescent centre (QC) and decreasing expression of *AtWOX5* and the auxin response reporter DR5 [17]. The *AUX ⁄LAX* gene family affects phyllotactic patterning and is needed to establish the embryonic root cell organization and plant embryogenesis in *Arabidopsis* [18, 19]. *PaLAX1*, from wild cherry (*Prunus avium*), promotes the absorption rate of auxin in cells and affects the distribution of free endogenous auxin [20]. *OsAUX1* controls the lateral root initiation, primary root and root hair elongation in rice [21, 22]. In *sorghum*, maize (*Zea mays*) and soybean (Glycine max), some *AUX/LAX* genes are in response to hormonal and abiotic stress at transcriptional level [23–25].

Among the auxin carriers, *PIN* family is extensively studied in *Arabidopsis*. The *PIN* family was first cloned and comprised of eight members in *Arabidopsis* [26]. The *PIN* family genes play crucial roles in various aspect of developmental processes, including root meristem patterning, root hair growth, lateral root development, vascular bundle differentiation, phototropism and embryo development [27–29]. PIN proteins are localised either on the plasma membrane (AtPIN1, −2, −3, −4 and −7) or in the endoplasmic reticulum (ER) (AtPIN5, AtPIN6 and AtPIN8). PIN proteins also play a vital role in both intracellular and intercellular auxin homeostasis [30, 31]. The PIN efflux transporter asymmetric localisation on the plasma membrane regulates the direction of the flow of auxin [32]. For example, AtPIN1 is asymmetrically localised on the basal rootward face of vascular cells [33]. The study of the PIN family has been expanded to other species not limited to *Arabidopsis*. In maize (*Zea mays*), two putative orthologues of *AtPIN1*, *ZmPIN1a* and *ZmPIN1b*, have been analysed involving in endosperm and embryonic development [34, 35]. In rice (*Oryza sativa*), as the closest orthologue of *AtPIN1*, *OsPIN1b* is been detected expressed in the roots, stem base, stem, leaves and young panicles [36, 37]. By analysis the phenotype of overexpression and RNAi lines, *OsPIN1b* may involve in auxin transport in primary and adventitious roots in rice [36]. The auxin transport from the shoot to the root–shoot junction is increased in *OsPIN2* overexpression plants. Overexpression of *OsPIN2* resulted in a larger tiller angle, a lowered plant height and an increased tiller number compared with the wild type [38]. A putative auxin efflux carrier of rice, *OsPIN3t*, is involved in the drought stress response and drought tolerance [39]. Three monocot-specific PIN genes from rice, *OsPIN9*, *OsPIN10a*, and *OsPIN10b*, are expressed at high level in adventitious root primordia and pericyclic cells at the stem base, suggesting that they might be involved in adventitious root development [37].

The ATP-binding cassette (ABC) superfamily contains more than 100 members in plants [40]. The subfamily B (ABCB), previously known as multidrug resistance (MDR)/phospho-glycoprotein (PGP) proteins, some of them are involved in auxin transport [41, 42]. Six members of ABCB transporters in *Arabidopsis* (AtABCB1, −4, −14, −15, −19 and −21) have been associated with auxin transport [41–44]. To date, *AtABCB1*, *AtABCB4* and *AtABCB19* are the best characterised *ABCB*s. Both AtABCB1 and AtABCB19 are involved in auxin efflux. AtABCB1 and AtABCB19 coordinate with AtPIN1 in long distance transport of auxin along the plant main axis, and regulate root and cotyledon development [45–47]. AtABCB4 and AtABCB21 function as an efflux and influx carrier that controls cellular auxin levels [44, 48]. AtABCB14 was first described as a malate importer modulating stomata aperture response to CO_2_ levels [49]. *AtABCB14* and *AtABCB15* are expressed in vascular tissues of primary stem by promoter::glucuronidase reporter assays. Anatomical alterations of the vascular tissue of the primary stem have been shown and IAA transport along the inflorescence is reduced in both *atabcb14* and *atabcb15* mutants, these results suggesting AtABCB14 and AtABCB15 might participate in auxin transport [43]. *OsABCB14*, a rice gene high homology with *AtABCB1*and *AtABCB19*, has been demonstrated as an auxin influx transporter, and its knockout mutants are insensitive to 2, 4-D and IAA. *OsABCB14* was found to be involved in iron homeostasis in rice [50].

Recently, auxin transporter genes have been studied throughout the plant kingdom, such as *Medicago sativa*, *Glycine max*, *Populus trichocarpa*, *Prunus avium*, *Oryza sativa*, *Sorghum bicolor*, and *Zea mays* [20, 23–25, 51]. However, little or nothing is known about the *LAX*, *PIN* and *ABCB* families in watermelon (*Citrullus lanatus*) to date. Watermelon is an important cucurbit crop and its output value accounted for more than 10% of the total output value of the vegetable industry in China. Watermelon seedling’s growth stops below 10 °C and cannot survive below the 1 °C [52]. Salinity and drought are the major environmental stresses in plant agriculture worldwide. Grafting is widely used to improve plants adaptation to biotic or abiotic stress [53, 54]. However, the expression of auxin transporter genes underlying grafting processes remains unclear. In this study, we provides comprehensive information on the *ClLAX*, *ClPIN* and *ClABCB* gene families and expression patterns of those genes exposed to salt, drought and cold stresses. The distinctive tissue-specific expression patterns of the *ClLAX*, *ClPIN* and *ClABCB* genes, and their differential responses to salt, drought and cold stresses are the molecular basis to increase abiotic stress tolerance in watermelon. Our studies also provide a new insight into the expression of *ClLAX*, *ClPIN* and *ClABCB* gene families at the phase of grafting.

## Methods

### Plant material, growth conditions and stress treatments

Watermelon “zaojia” was selected in this study. Seeds were sown in perlite beds after sterilized with 10% sodium hypochlorite for 30 min. Seedlings at the two-leaf were irrigated by half-strong Hoagland solution (pH5.6). The growth conditions were as follows: a 12 h photoperiod under fluorescent light (600 μE m^2^ s^−1^) at with 60% relative humidity, and temperature of 28/18 °C (day/night). A month old seedlings were used for stress treatment.

For auxin treatment, the roots of watermelon seedlings were soaked in half-strong Hoagland nutrient solution containing 100 μM IAA. For salt stress experiment, the roots of seedlings were immersed in nutrient solution containing 200 mM NaCl. For drought stress experiment, the roots of seedlings were immersed in nutrient solution containing 20% (W/W) PEG6000 (Polyethylene glycol). For cold treatment, seedlings were transferred to a 4 °C growth chamber. Then root and shoot samples of watermelon seedlings at different treatment time points were harvested. For graft experiment, watermelon plants when cotyledon had expanded were grafted onto squash performed by ‘top approach grafting’ method. Experiment was repeated for 3 times with similar results.

For tissue-specific expression analysis, roots, stems, leaves, and cotyledons samples were harvested from two-leaf stage; for flower samples, flowers were harvested at 2 d after opening.

### Identification of *ClLAX*, *ClPIN* and *ClABCB* auxin transporter family genes in watermelon

The sequences of *ClLAX*, *ClPIN* and *ClABCB* were collected by homology screening against Cucurbit Genomics Database (http://www.icugi.org/cgi-bin/ICuGI/index.cgi) (version 1). The known sequences of *AtLAX*, *AtPIN* and *AtABCB* were used as queries. The hidden Markov model profiles were used to identify LAX, PIN and ABCB proteins from the proteome of watermelon. Pfam 01490 (Transmembrane amino acid transporter protein) was used for ClLAX proteins identification; Pfam 03547 (Membrane transport protein) was used for ClPIN proteins identification; Pfam 00005(ABC transporter) and Pfam 00664 (ABC transporter transmembrane region) were used for ClABCB proteins identification. Protein molecular weight and isoelectric point were predicted by DNAstar tool (http://www.dnastar.com/). The transmembrane helices of ClLAX, ClPIN and ClABCB proteins were predicted by TMHMM2 Software (http://www.cbs.dtu.dk/services/TMHMM/).

### Genome distribution, phylogenetic tree building and intron/exon structure

The chromosomal location data of *ClLAX*, *ClPIN* and *ClABCB* family genes were obtained from Cucurbit Genomics Database. A map of the distribution of *ClLAX*, *ClPIN* and *ClABCB* family genes was drawn based on their chromosomal position. The alignment file of LAX, PIN and ABCB family proteins in watermelon and Arabidopsis was generated by ClustalW program with the default parameters. The amino acid sequences of AtLAX, AtPIN and AtABCB proteins were obtained from Yue et al. [24]. Phylogenetic tree was performed by MEGA6.0 (http://www.megasoftware.net/) using the neighbor-joining (NJ) method with the p-distance and complete deletion parameters. Exon-intron structure of *ClLAX*, *ClPIN* and *ClABCB* family genes were employed by Gene Structure Display Server (GSDS) tool (http://gsds.cbi.pku.edu.cn/).

### Quantitative real time-polymerase chain reaction PCR (qRT-PCR)

Total RNA were extracted from 0.1 g of samples using MiniBEST Plant RNA Extraction Kit (code: 9769, TAKARA, Japan) according to the manufacturer’s instruction. The primers sequences of qRT-PCR are listed in Additional file 1: Table S1. Quantitative RT-PCR was performed on LightCycler480 instrument (Roche) according to the manufacturer’s instructions. The *ClACTIN* (*Cla004014*) was used as internal standards basing on the comparative cycle threshold (2^-ΔΔCt^) values. Heat map was performed by MeV software using the average Ct value to visualize the tissues-specific expression data. All the expression analyses were carried out with three biological repeats.

## Results

### Genome-wide identification of *ClLAX*, *ClPIN* and *ClABCB* genes in watermelon

In the present study, we used the AUX/LAX, PIN and ABCB full-length protein sequences from *Arabidopsis* as BLAST queries to search Cucurbit Genomics Database (http://www.icugi.org/). Four hidden Markov model profiles (Pfam 01490, Pfam 03547, Pfam 00005 and Pfam 00664) were used to identify the ClLAX, ClPIN and ClABCB proteins. Totally seven *ClLAX* genes, 11 *ClPIN* genes and 15 *ClABCB* genes were identified. We named them based on their order on the chromosomes. Information on *ClLAX*, *ClPIN* and *ClABCB* gene families, including gene names, locus ID, open reading lengths, exon numbers, chromosome locations and deduced polypeptide parameters, were listed in Table 1.

**Table 1:** Information on *ClLAX*, *ClPIN* and *ClABCB* genes and properties of the deduced proteins in watermelon (*Citrullus lanatus*)

Gene	Locus ID	ORF lengh (bp)	No. of extrons	Chromosome No.	Deducted polypeptid			No. of transmembrane
					Length (aa)	Ml wt (Da)	pI	
*CILAX1*	Cla015837	1470	8	Chr2	489	54960.23	8.927	10
*CILAX2*	Cla020298	1461	8	Chr2	486	54841.35	9.258	10
*CILAX3*	Cla018110	1326	7	Chr4	441	49895.36	8.204	8
*CILAX4*	Cla004339	1467	7	Chr7	488	54875.07	7.804	10
*CILAX5*	Cla017975	1437	7	Chr10	478	53841.09	8.331	10
*CILAX6*	Cla006581	1329	8	Chr11	442	49504.24	8.485	8
*CILAX7*	Cla000681	1443	8	chr0	480	54063.26	8.532	10
*CIPIN1*	Cla003909	1926	6	chr1	641	69903.01	7.439	9
*CIPIN2*	Cla010530	1041	5	chr2	346	37608.62	7.982	8
*CIPIN3*	Cla010532	564	3	chr2	187	19891.53	5.453	2
*CIPIN4*	Cla012098	1848	6	chr4	615	65610.47	7.899	8
*CIPIN5*	Cla018455	1860	6	chr4	619	67586.08	9.088	9
*CIPIN6*	Cla018871	1896	6	chr6	631	69221.59	9.178	5
*CIPIN7*	Cla018924	1824	7	chr6	607	66418.92	9.149	9
*CIPIN8*	Cla011709	1005	5	chr7	334	36418.55	10.852	2
*CIPIN9*	Cla011708	675	1	chr7	224	25017.54	7.190	5
*CIPIN10*	Cla015026	1449	5	chr9	482	53345.51	9.198	6
*CIPIN11*	Cla017028	1092	6	chr10	363	40031.79	9.761	7
*CIABCB1*	Cla009733	3765	12	chr1	1254	135542.15	7.431	10
*CIABCB2*	Cla006778	3675	10	chr2	1224	134618.81	7.096	11
*CIABCB3*	Cla006779	3708	10	chr2	1235	135741.22	7.262	11
*CIABCB4*	Cla010534	3897	12	chr2	1298	139885.34	8.658	10
*CIABCB5*	Cla011266	3504	9	chr3	1167	127972.37	9.286	8
*CIABCB6*	Cla001708	3750	7	chr5	1249	137870.07	8.638	12
*CIABCB7*	Cla007439	3906	12	chr5	1301	141742.47	7.289	11
*CIABCB8*	Cla010011	3780	7	chr5	1259	137952.57	8.756	9
*CIABCB9*	Cla015527	4368	9	chr9	1455	159168.71	8.788	12
*CIABCB10*	Cla016230	3612	12	chr9	1203	131803.14	8.908	10
*CIABCB11*	Cla010337	3753	10	chr9	1250	136474.22	8.189	10
*CIABCB12*	Cla010365	3750	7	chr9	1249	135971.07	8.733	9
*CIABCB13*	Cla004699	4200	11	chr9	1399	155709.89	6.722	12
*CIABCB14*	Cla017800	4080	9	chr10	1359	148875.01	6.949	11
*CIABCB15*	Cla022922	3633	11	chr11	1210	130903.31	8.198	8

The sizes of the ORF for the *ClLAX* genes ranged from 1326 bp (*ClLAX3*) to 1470 bp (*ClLAX1*), and the sizes of the corresponding proteins were between 441 and 489 amino acids. The molecular masses of ClLAX protein varied from 49.5 kDa (ClLAX6) to 54.96 k Da (ClLAX1). The predicted isoelectric points ranged from 7.804 (ClLAX4) to 9.258 (ClLAX2). The number of transmembrane of ClLAX proteins predicted by TMHMM2 software was between 8 and 10. The sizes of the ORF for the *ClPIN* genes ranged from 564 bp (*ClPIN3*) to 1926 bp (*ClPIN1*). The sizes of the corresponding proteins were between 187 and 641 amino acids. The molecular masses of ClPIN protein varied from 19.89 kDa (ClPIN3) to 69.9 k Da (ClPIN1). The predicted isoelectric points varied from 5.453 (ClPIN3) to 10.852 (ClPIN8). The sizes of the ORF for the *ClABCB* genes ranged from 3504 bp (*ClABCB5*) to 4368 bp (*ClABCB9*), and the sizes of the corresponding proteins are between 1167 and 1455 amino acids. The molecular masses of ClABCB protein varied from 127.97 kDa (ClABCB5) to 159.17 kDa (ClABCB9). The predicted isoelectric points varied from 6.722 (ClABCB13) to 9.286 (ClABCB5).

### Chromosomal distribution of *ClLAX*, *ClPIN* and *ClABCB* genes

**Fig. 1:**
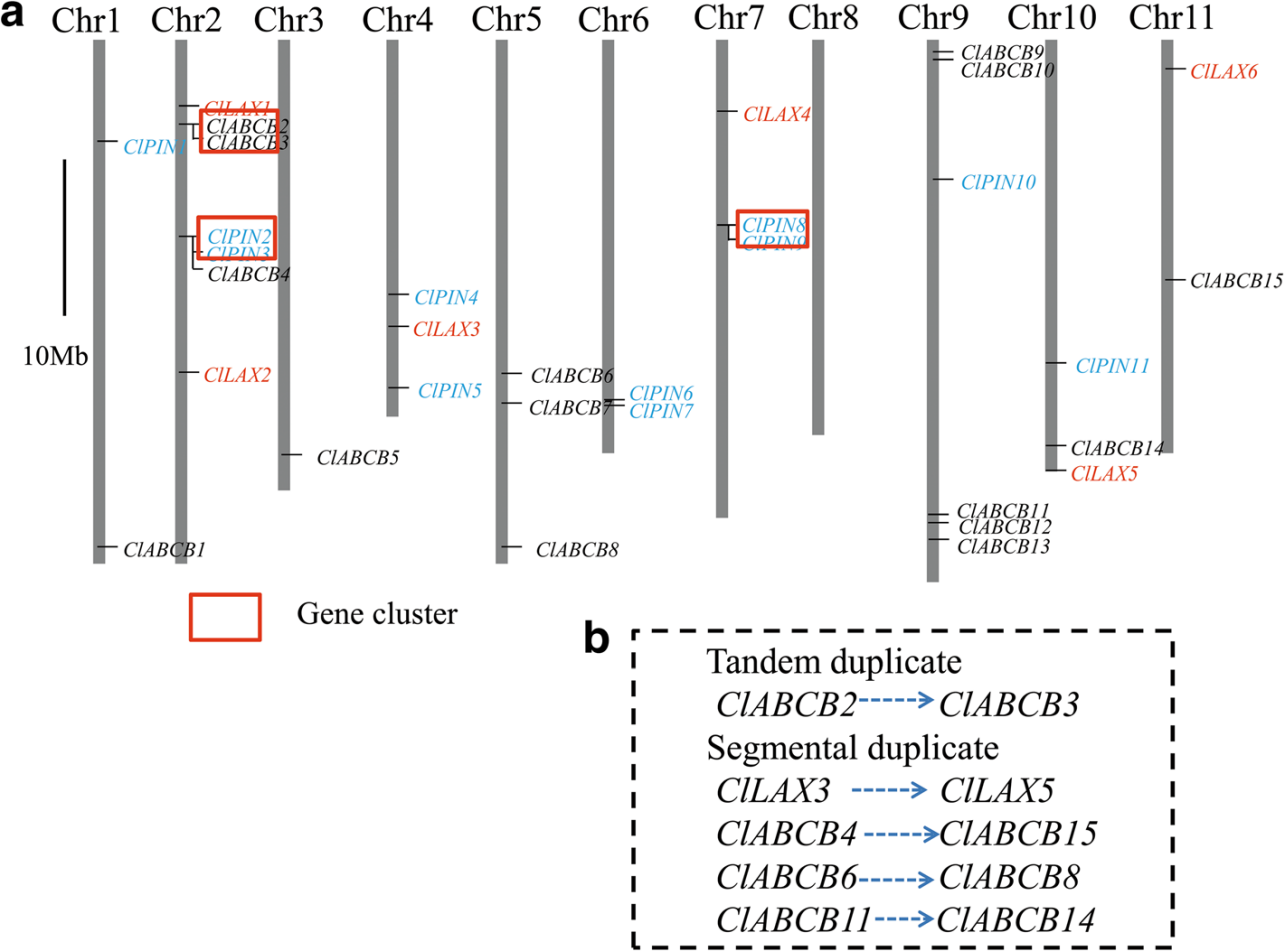
Chromosomal distributionsand expansion patterns of *ClLAX*, *ClPIN* and *ClABCB* family genes in watermelon. **a** The genome visualization tool SyMAP Synteny Browser was employed to analyze the watermelon genome. Watermelon chromosomes were arranged in blocks. Seven *ClLAX* genes, 11 *ClPIN* genes and 15 *ClABCB* genes were mapped by locus. *Red boxes* presented the gene clusters. **b** Tandem duplicate *ClABCB2/ClABCB3* and Four pairs of segmental duplicates *ClABCB4*/*ClABCB13*, *ClABCB6*/*ClABCB8*, *ClABCB11*/*ClABCB14* were showed with dotted arrows. Scale bar represents 10 Mb

Based on position of *ClLAX*, *ClPIN* and *ClABCB* genes on the watermelon chromosomes, we mapped all 33 genes of *ClLAX*, *ClPIN* and *ClABCB* family on chromosomes (Fig. 1a, Table 1). The 33 genes were unevenly distributed on 10 out of the 11 watermelon chromosomes. Among 33 genes, not a single gene was located on chromosome 8. Chromosome 3 only contained one gene. Two genes were located on chromosome 1, 6 and 11, respectively. Three genes were distributed on each of chromosomes 4 and 7. Seven genes were located on chromosome 2 (Fig. 1a). In many plants, including *S.bicolor*, *Arabidopsis*, *G.max*, *O.sativa*, some of the auxin transporter genes were clustered. Three small gene clusters were identified in accord with the definition of gene clusters [55]. Two gene clusters were distributed on chromosome 2 (Fig. 1a). The other one was distributed on chromosome 7. The first gene cluster contained two *ClABCB* genes (*ClABCB2* and *ClABCB3*). The second gene cluster contained two *ClPIN* genes (*ClPIN2* and *ClPIN3*). The third gene cluster contained two *ClPIN* genes (*ClPIN8* and *ClPIN9*).

Gene duplication is the main contributor to evolutionary momentum [56]. The duplication patterns of *ClLAX*, *ClPIN* and *ClABCB* families including tandem and segmental duplications were analyzed to find the expansion of *ClLAX*, *ClPIN* and *ClABCB* gene families during the evolutionary momentum. Tandem duplication was observed between *ClABCB2* and *ClABCB3* (Fig. 1b). *ClLAX3*/*ClLAX5* gene pairs share high similarity in protein sequences (Additional file 2: Table S2), and were lactation on different chromosomes, indicating that they were segmental duplicated gene pair. Three segmental duplications occurred in the *ClABCB* gene family: *ClABCB4*/*ClABCB15*, *ClABCB6*/*ClABCB8* and *ClABCB11*/*ClABCB14* (Fig. 1b).

### Phylogenetic relationship analysis of the *ClLAX*, *ClPIN* and *ClABCB* family genes

**Fig. 2:**
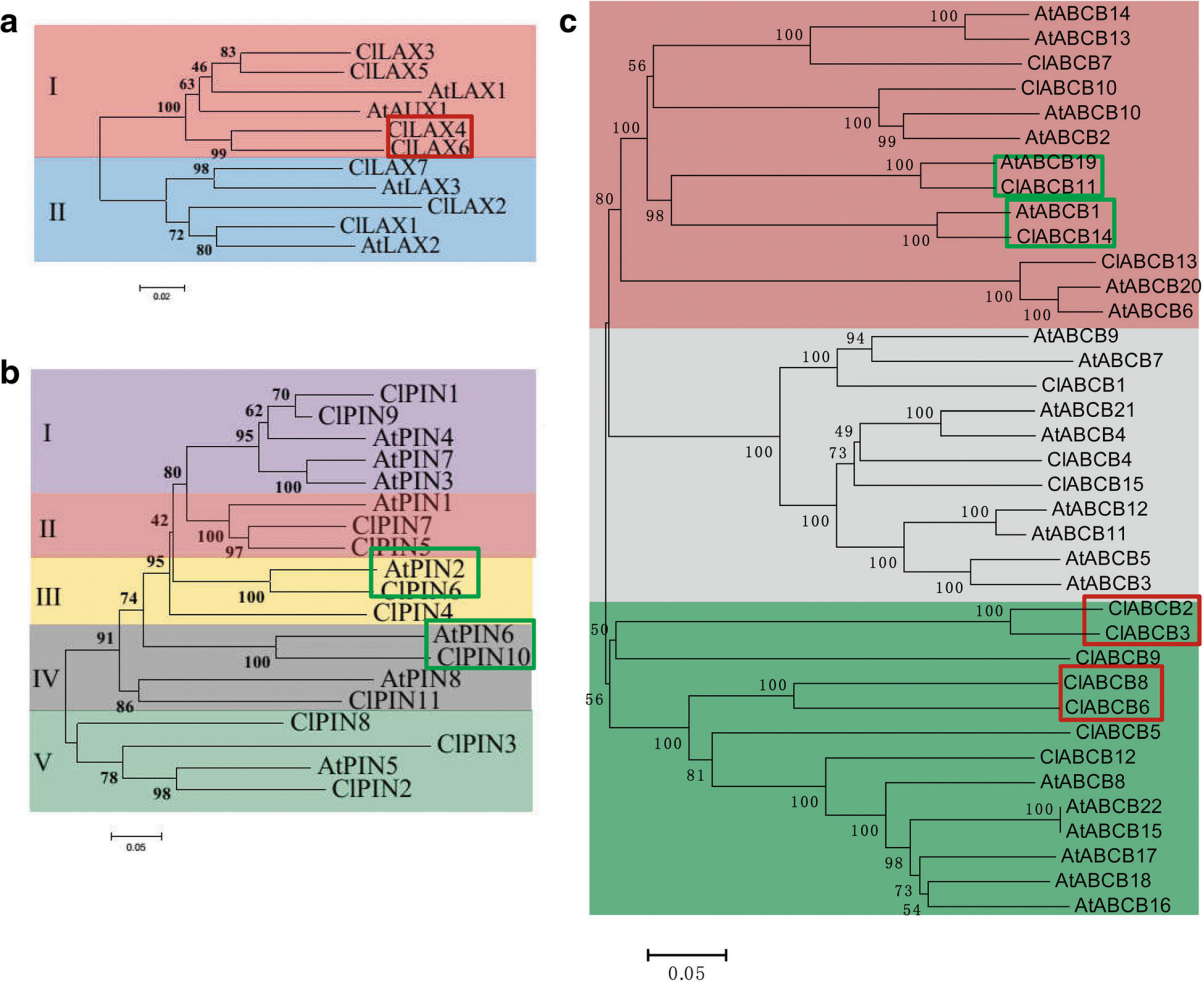
Phylogenetic relationship analysis of LAX (**a**), PIN (**b**) and ABCB (**c**) family between watermelon and *Arabidopsis*. All branches were marked with bootstrap values. Inventory of AtLAX, AtPIN families is based on TAIR databases. The paralogous genes between watermelon and Arabidopsis were indicated by *green boxes*. The orthologous genes within watermelon were indicated by *red boxes*

Many studies revealed the biological functions of the auxin transporter genes in *Arabidopsis* [11, 29, 42]. Investigation of the evolutionary relationships of three kinds of auxin transporter proteins between watermelon and *Arabidopsis* helps us to understand the possible biological functions of these auxin carriers in watermelon. Multiple protein sequence alignments of full-length amino acid sequence were carried out using the MEAG6.0 software for phylogenetic analysis with the neighbour-joining method. A total of 11 AUX/LAX proteins, including 7 ClLAX proteins and 4 AtLAX proteins were used to build a phylogenetic tree (Fig. 2a). The *LAX* family genes could be divided into two subfamilies (subfamily I and subfamily II). Six of them belong to subfamily I (*ClLAX3*, *4*, *5*, *6*, *AtAUX1* and *AtLAX1*). A paralogue gene pair existed in the watermelon *LAX* family: *ClLAX4*/*ClLAX6*. A total of 19 PIN proteins, including 11 ClPIN proteins and 8 AtPIN proteins were used to construct a phylogenetic tree (Fig. 2b). All the PIN family could be grouped into five subfamilies (subfamily I, -II, -III, -IV and-V). Two PIN orthologue gene pairs existed between watermelon and *Arabidopsis*: *ClPIN6*/*AtPIN2* and *ClPIN10*/*AtPIN6*. A total of 37 ABCB proteins, including 15 ClABCB proteins and 22 AtABCB proteins were used to construct a phylogenetic tree (Fig. 2c). All the ABCB families could be classified into three subfamilies (subfamily I, subfamily II and subfamily III). Two ABCB orthologue gene pairs were existed between watermelon and *Arabidopsis*: *ClABCB14/AtABCB1* and *ClABCB11/AtABCB19*. Two paralogue gene pair occurred in the watermelon *ABCB* family: *ClABCB2/ClABCB3* and *ClABCB6/ClABCB8*.

### Analysis of tissue-specific expression and gene structure of *ClLAX*, *ClPIN* and *ClABCB* family genes

**Fig. 3:**
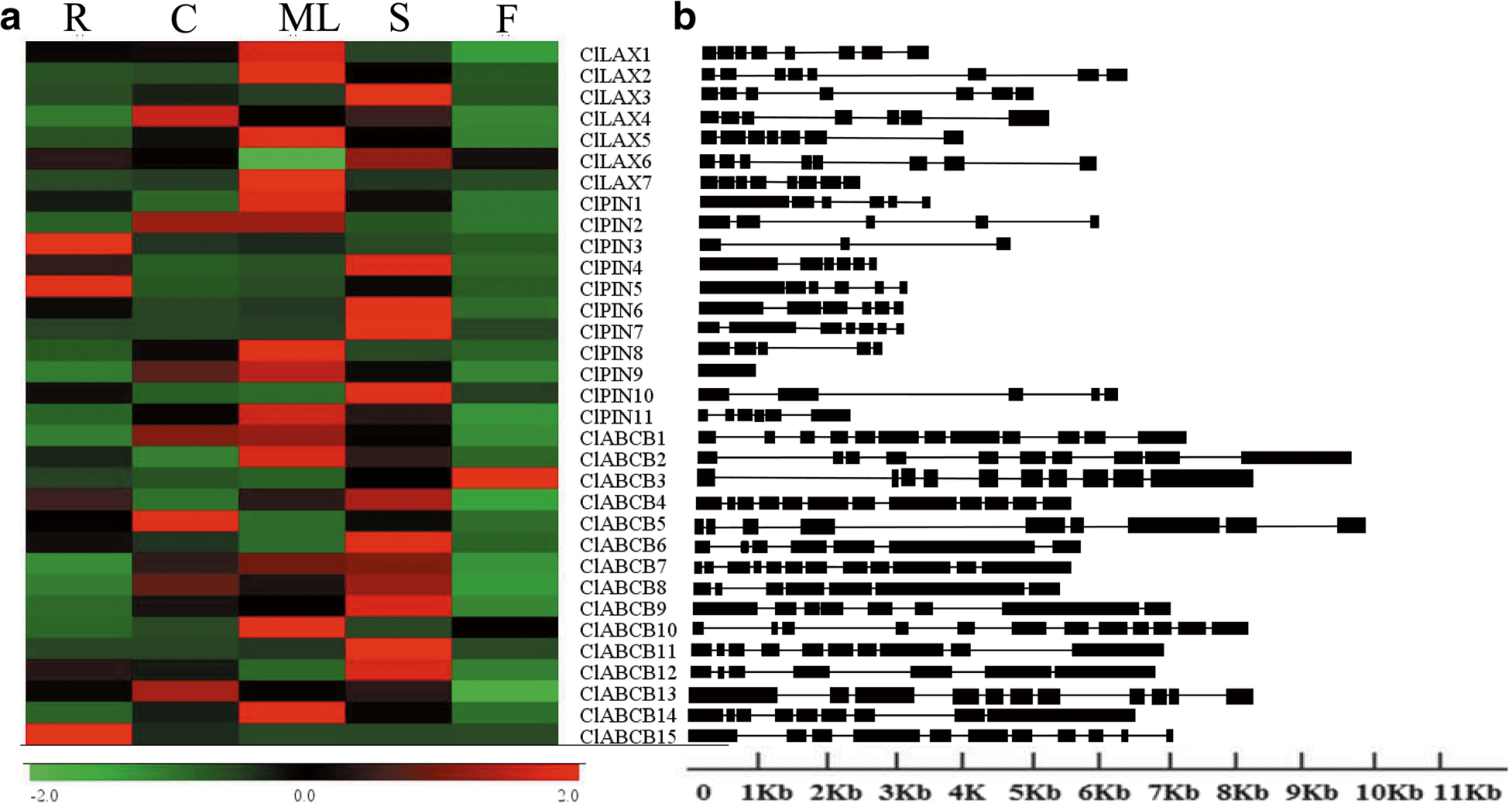
Analysis of tissues-specific expressions (**a**) and exon-intron structures (**b**) of *ClLAX*, *ClPIN* and *ClABCB* genes. R: root; C: cotyledon; ML: mature leaf; S: shoot; F: flower. Levels of different colours were shown on expression scale of each *ClLAX*, *ClPIN* and *ClABCB* genes. The exons were indicated by *black boxes*; the introns were indicated by *black lines*

To elucidate the biological roles of different members of the *ClLAX*, *ClPIN* and *ClABCB* family in watermelon, the expression of *ClLAX*, *ClPIN* and *ClABCB* genes was investigated in different tissues performed by quantitative real-time polymerase chain reaction (qRT-PCR). Total RNA was extracted from the roots, cotyledons, mature leaves, stems and flowers of watermelon. All transcripts of *ClLAX*, *ClPIN* and *ClABCB* family genes were detected in the selected tissues. Most of the *ClLAX*, *ClPIN* and *ClABCB* genes showed different tissue-specific patterns across the five tissues. As shown in Fig. 3a, the transcriptional level of the *ClLAX* family gene was the highest in the mature leaves and the lowest in the flowers. *ClPIN3* and *ClPIN5* were highly expressed in roots. *ClPIN1*, *ClPIN8* and *ClPIN11* showed the highest level of expression in mature leaves. Most of *ClPIN* genes were weakly expressed in the flowers. *ClABCB3* was much more highly expressed than any other *ClABCB* genes in the flowers. The level of expression of *ClABCB* genes was much higher in the stems than in the flowers. All the expression levels of the *ClLAX*, *ClPIN* and *ClABCB* family genes in five tissues are listed in Additional file 3: Table S3.

Gene structure analysis of the *ClLAX*, *ClPIN* and *ClABCB* family genes was revealed by comparing the coding sequences with genomic DNA sequences. The exon–intron structures of the three family genes revealed variations (Fig. 3b). The gene exon number of *ClLAX* genes was either seven or eight. The number of exons in *ClPIN* genes varied from one (*ClPIN9*) to seven (*ClPIN7*). The number of exons in *ClABCB* genes varied from 7 to 12.

### Expression profiles of *ClLAX, ClPIN* and *ClABCB* family genes upon IAA treatment

**Fig. 4:**
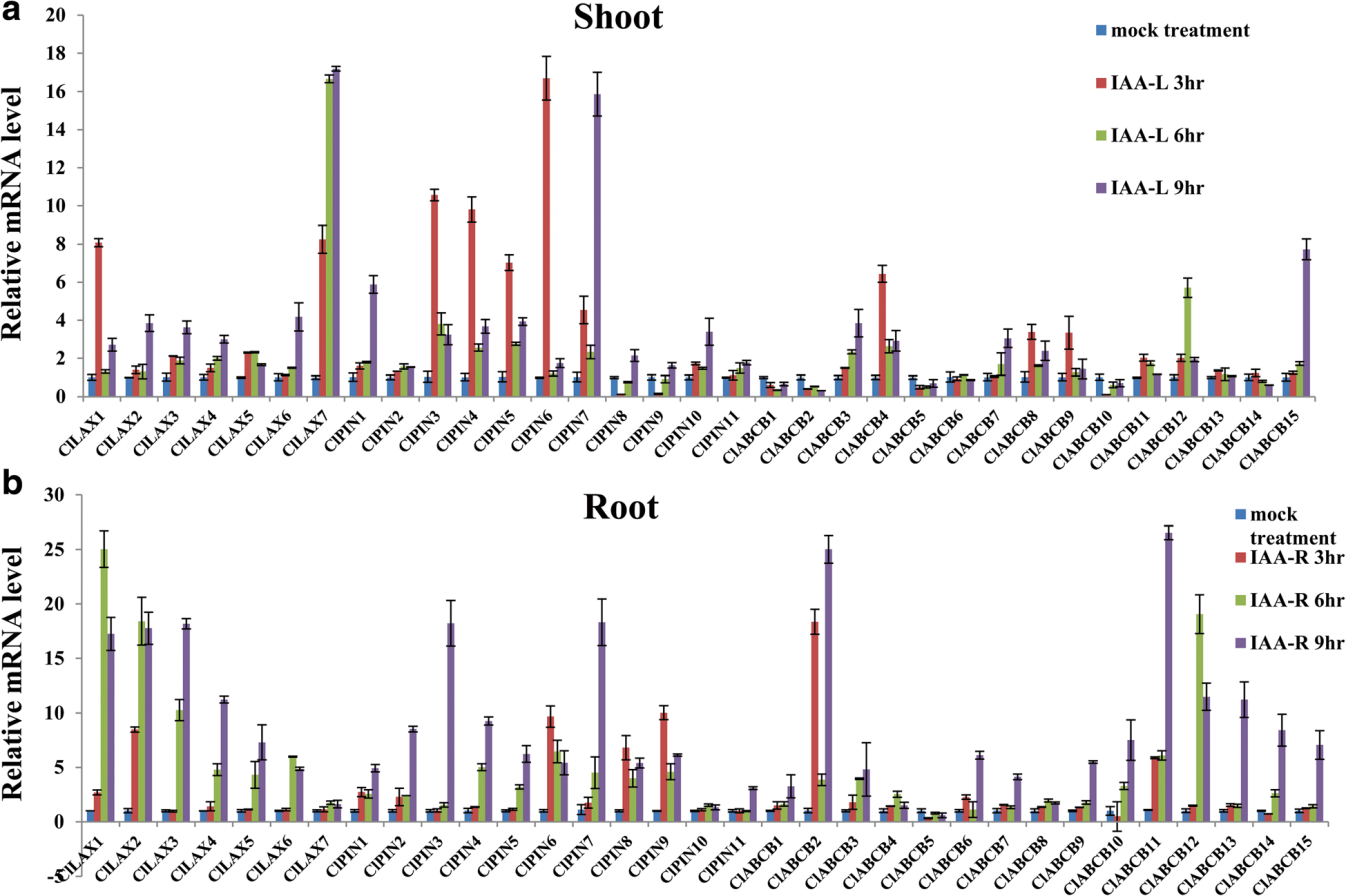
Expression profiles analysis of *ClLAX*, *ClPIN* and *ClABCB* family genes under IAA treatment in shoots (**a**) and roots (**b**). Total RNA was extracted from the shoots and roots of 3-week-old watermelon seedlings for expression analysis. The histogram shows the relative expression levels of *ClLAX*, *ClPIN* and *ClABCB* genes under IAA (10 μM) treatment compared to the untreated expression level. The *ClACTIN* (*Cla004014*) was used as internal standards. The relative expression levels were normalized to a value of 1 in mock seedlings

Auxin regulating plant growth and development depends mainly on auxin transporter to regulate auxin relocation and homeostasis [23, 24]. Exogenous auxin treatment could accelerate or block the endogenous auxin transport between different tissues [23, 57]. To investigate whether the auxin transporters in watermelon were regulated by auxin, the expression profiles of *ClLAX*, *ClPIN* and *ClABCB* genes under 10 μM IAA for 9 h in the shoots and roots were analysed by qRT-PCR (Fig. 4). Total RNA was isolated from the shoots and roots of mock seedlings or IAA-treated seedlings at different time points (6, 12 and 24 h). Our data suggested that most *ClLAX*, *ClPIN* and *ClABCB* genes were auxin responsive genes. The majority of these genes were differentially regulated by IAA at the transcriptional level. IAA treatment increased the expression levels of *ClLAX1*, *−7*, *ClPIN3*,*−4*, *−5*, *−6*, *−7* and *ClABCB4* in the shoots more than five-fold. On the contrary, *ClABCB1*, *−2*,*−5*,*−10* and *−14* expression levels were downregulated in the shoots after IAA treatment (Fig. 4a). Most of the auxin transporter genes were upregulated after IAA treatment in the roots (Fig. 4b). IAA treatment upregulated the expression levels of *ClLAX1*, *−2*, *−3*, *ClPIN3*, *−7*, *ClABCB2*, *−10* and *−12* more than15-fold in the roots. In both the roots and shoots, the expression of *ClABCB5* was down-regulated by IAA treatment.

### Expression Profiles of *ClLAX, ClPIN* and *ClABCB* family genes under abiotic stresses

**Fig. 5:**
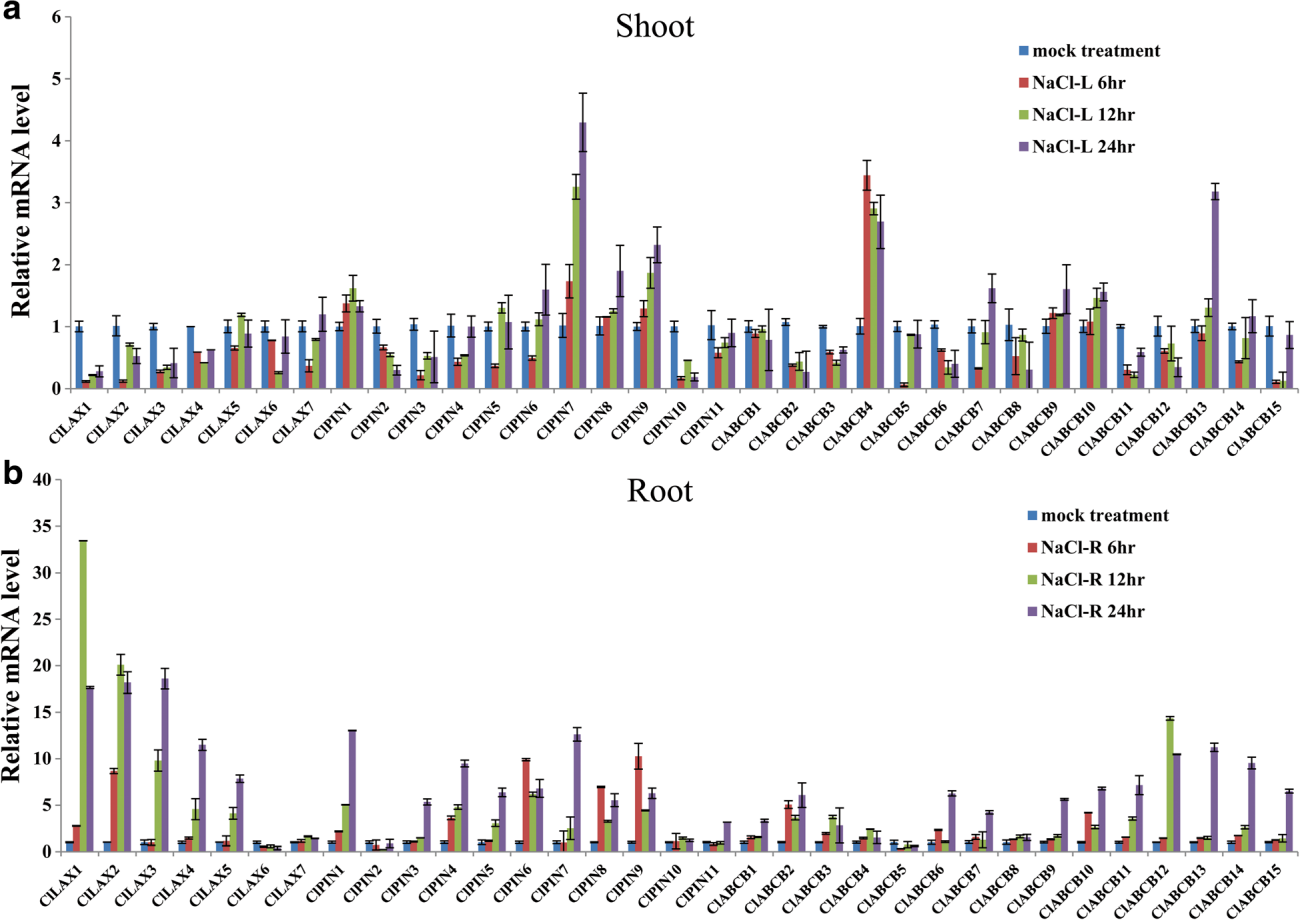
Expression profiles analysis of *ClLAX*, *ClPIN* and *ClABCB* family genes in response to salt stress in both shoot (**a**) and roots (**b**). Expression levels of *ClLAX*, *ClPIN* and *ClABCB* genes were analysed by qRT-PCR using 3-week-old watermelon seedlings, which were treated with 200 μM NaCl for 24 h. The relative expression levels were normalized to a value of 1 in mock seedlings

**Fig. 6:**
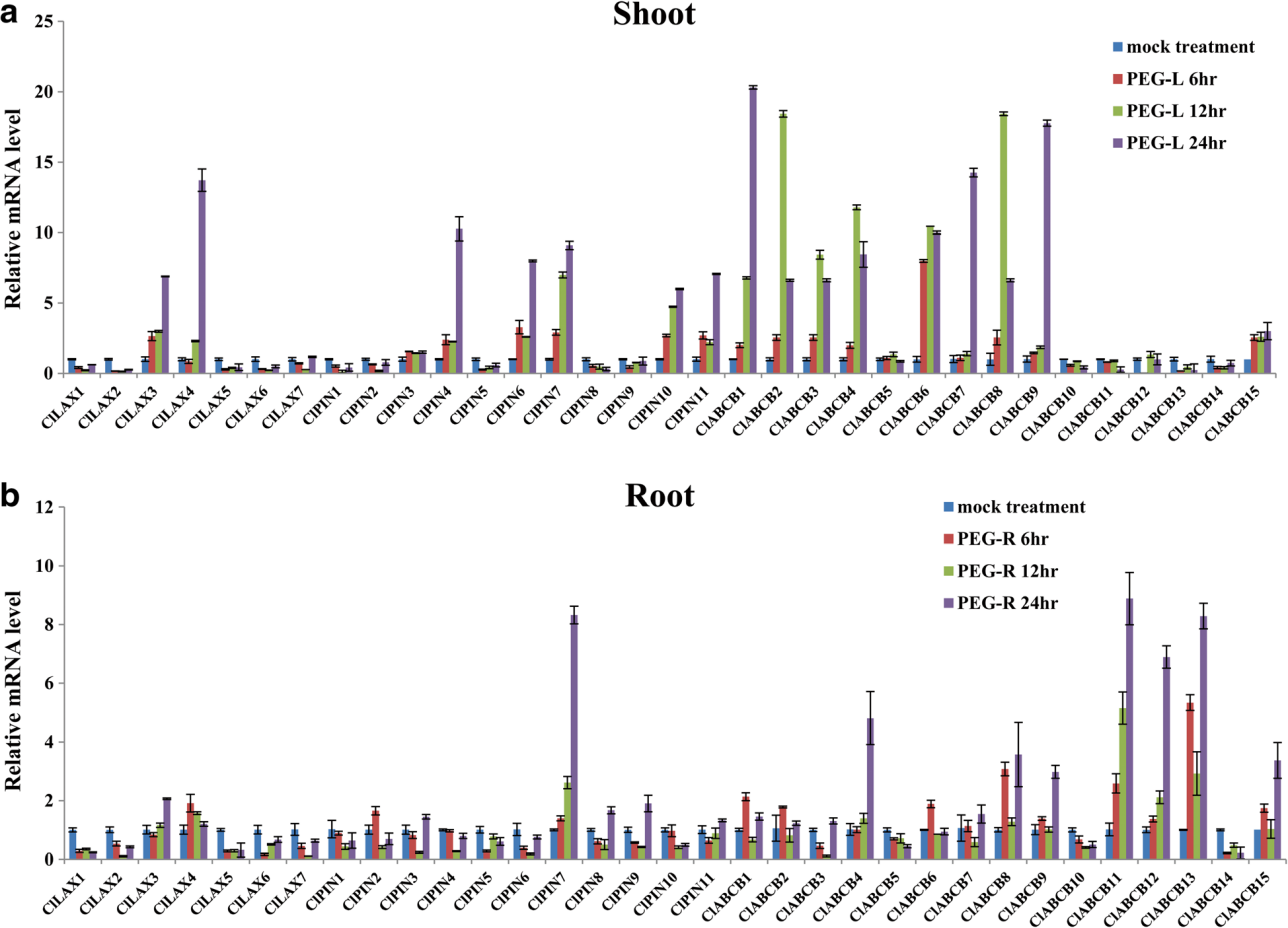
Expression profiles analysis of *ClLAX*, *ClPIN* and *ClABCB* family genes in response to drought stress. Expression levels of *ClLAX*, *ClPIN* and *ClABCB* genes were analysed by qRT-PCR in both shoot (**a**) and roots (**b**) of 3-week-old watermelon seedlings, which were treated with 20% (W/V) Polyethylene glycol (drought) for 24 h. The relative expression levels were normalized to a value of 1 in mock seedlings

**Fig. 7:**
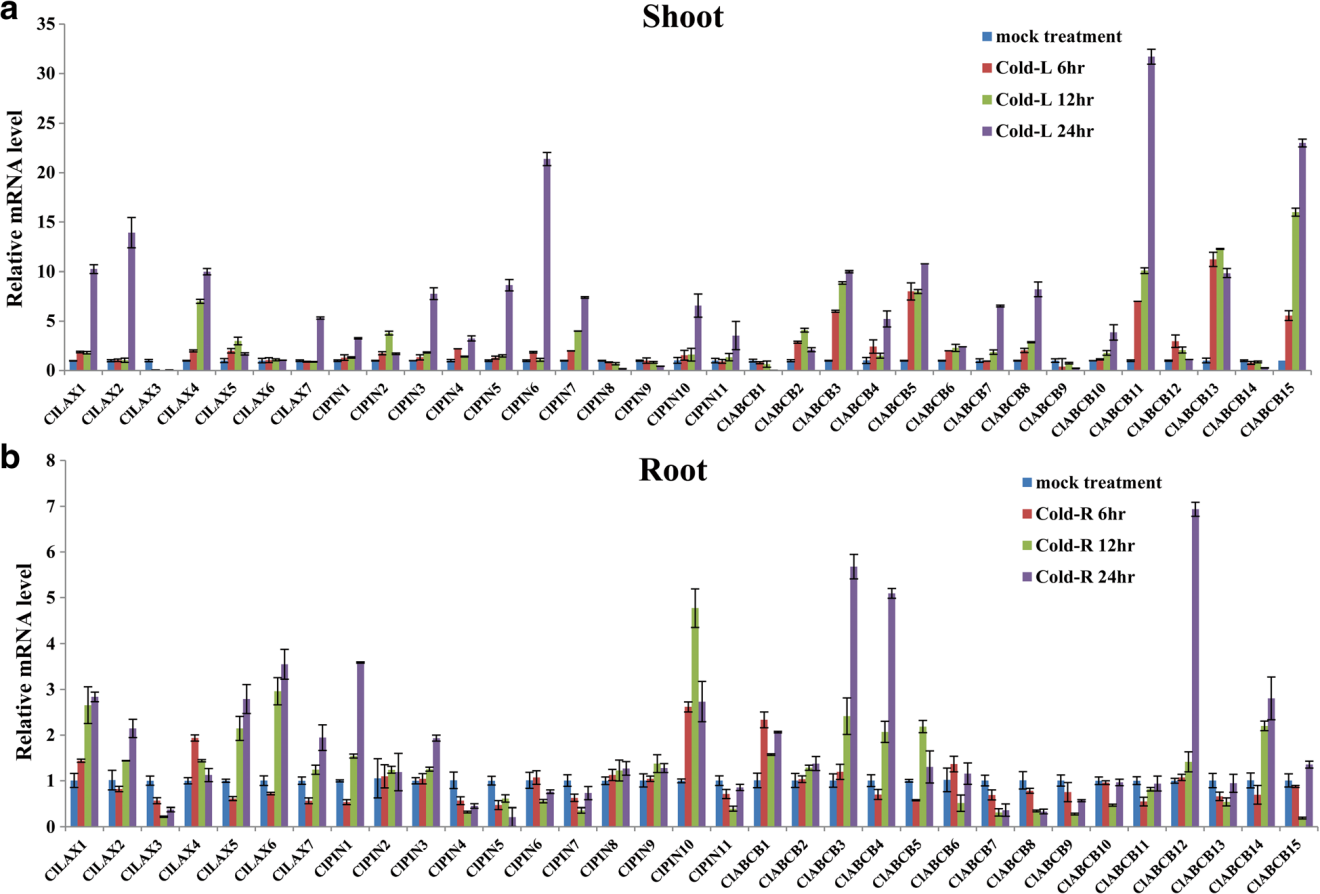
Expression profiles analysis of *ClLAX*, *ClPIN* and *ClABCB* family genes in response to cold stress in both shoot (**a**) and roots (**b**). The histogram shows the relative expression levels of *ClLAX*, *ClPIN* and *ClABCB* genes under 4 °C treatment compared to the untreated expression levels, which were normalized to a value of 1

Watermelon is one of the most drought and salinity sensitive cucurbit crops. Its yield is significantly influenced by these abiotic stresses such as drought, salinity and cold [52]. Many studies showed that auxin is involved in stress response, and a quantity of auxin transporter genes are associated with abiotic stress responses. To investigate whether *ClLAX, ClPIN* and *ClABCB* genes are involved in abiotic stress response, the expressions levels of 33 auxin transporter genes were investigated under salinity (NaCl), drought (PEG) and cold (4 °C) treatment using qRT-PCR (Figs. 5, 6 and 7). Untreated seedlings growing under normal condition were used as control.

Different *ClLAX, ClPIN* and *ClABCB* expression patterns were observed in the roots and shoots when they were treated with the abiotic stress treatment. The majority of the *ClLAX, ClPIN* and *ClABCB* genes were downregulated in the shoots after 200 μM NaCl treatment (Fig. 5a). However, most of the *ClLAX*, *ClPIN* and *ClABCB* genes were upregulated in the roots after NaCl treatment (Fig. 5b). Only the expression of *ClLAX6*, *ClPIN2*, and *ClABCB5* was inhibited by NaCl treatment in the roots. Half of *ClLAX, ClPIN* and *ClABCB* genes were upregulated in the shoots under PEG treatment (Fig. 6a). *ClLAX3*, *−4*, *ClPIN4*, *−6*, *−7*, *−10*, *−11, ClABCB1*, *−2*, *−3*, *−4*, *7*, *−8*, *−9* and *−15* were induced (>5 fold) by PEG treatment after 24 h in the shoots. However, only *ClPIN7*, *ClABCB12* and *ClABCB13* were induced (>5 fold) by PEG treatment after 24 h in the roots (Fig. 6b). Half of *ClLAX, ClPIN* and *ClABCB* genes were upregulated in shoots under cold treatment for 24 h (Fig. 7a). The expression of *ClLAX2*, *ClPIN4*, *−5*, *−7*, *ClABCB7*, *−8*, *−9*, *−11* and *−13* were down-regulated in the roots by cold treatment (Fig. 7b).

### Expression profiles of *ClLAX*, *ClPIN* and *ClABCB* family genes in grafting response

**Fig. 8:**
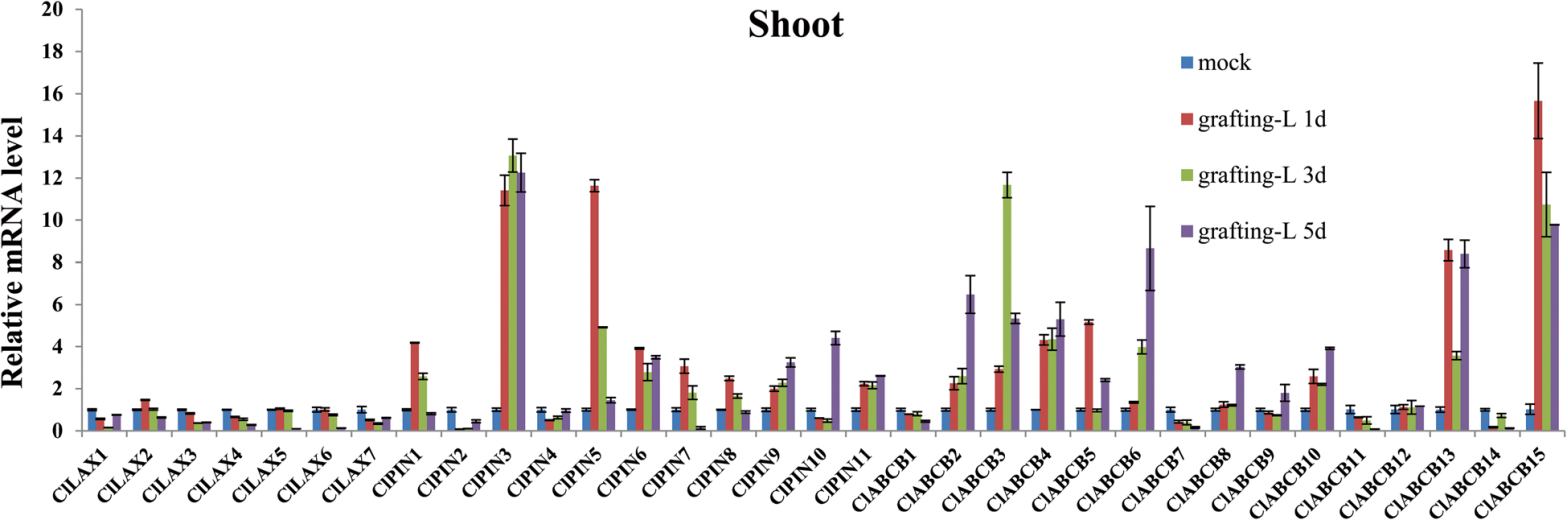
qRT-PCR analysis of *ClLAX*, *ClPIN* and *ClABCB* family genes in grafting-responsive

Grafting is an ancient technique that is widely used in agriculture practices to improve productivity and stress resistance [53]. Auxin can increase the activity of cell division and wound healing in cut *Arabidopsis* inflorescence stems. However, the molecular mechanisms of auxin involved in these processes remain largely unclear. To investigate whether auxin transporter genes from watermelon are involved in grafting response, we analysed the expression profiles of *ClLAX*, *ClPIN* and *ClABCB* during grafting for 5 days in the shoots (Fig. 8). The data indicated that most of the *ClLAX* genes were downregulated and most of the *ClPIN* genes were upregulated in the shoots during grafting. Only *ClABCB1*, *−7*, *−11* and *−4* were down-regulated. The rest of the *ClABCB* family genes were significantly upregulated.

## Discussion

Auxin, as a key regulator of plant growth and development through polar auxin transport, is involved in response to environmental stress [2, 3]. In recent years, the molecular mechanism of auxin transport has been gradually elucidated in *Arabidopsis*. On the basis of the function in auxin transport, auxin transport proteins are divided into three major families. They were AUX ⁄LAX influx carriers, PIN efflux carriers and ABCB efflux ⁄conditional transporters. With the publication of the *C.lanatus* genome [58], we have a further understanding of the molecular mechanism of auxin transport in watermelon. In the current research, we identified 33 auxin transporter genes in watermelon and concentrated on the expression profiles of *ClLAX*, *ClPIN* and *ClABCB* genes to elucidate how the auxin transporters were involved in watermelon responses to salt, drought or cold stresses and the phase of grafting.

### Characterisation of *ClLAX*, *ClPIN* and *ClABCB* genes in watermelon

Watermelon (*Clanatus*), an important vegetable crop with 425 Mb genome size, accounts for approximately 7% of the agricultural area worldwide based on the statistics from Food and Agriculture Organization. Our study characterized the complete *ClLAX*, *ClPIN* and *ClABCB* family genes in watermelon. The numbers of *ClLAX* and *ClPIN* family genes in watermelon were more than those in *Arabidopsis*. The number of *LAX* genes in watermelon is around twice the number in *Arabidopsis*. The number of *ClABCB* family genes in watermelon is less than that in *Arabidopsis. Arabidopsis* homologous genes are widely existed in watermelon genome. The relatively similar protein sequence identities of the LAX, PIN and ABCB proteins between watermelon and *Arabidopsis* implied that all these genes originated from one or more common genes [59]. Two sister pair genes were identified as orthologue genes between watermelon and *Arabidopsis* in *PIN* family with bootstrap values ≥ 99%. Two sister pair genes were identified in *ABCB* family between watermelon and *Arabidopsis*. However, no orthologue gene pairs were identified in *LAX* family between watermelon and *Arabidopsis* (bootstrap value ≥ 99%). ClLAX, ClPIN and ClABCB proteins contain multiple transmembrane helices, which are similar to the conserved structure of auxin transport protein from *Arabidopsis* [37, 57]. The ClLAX proteins only contain one group of transmembrane helices, and there is no variable middle hydrophilic region in ClLAX proteins (Additional file 4: Figure S1). Two groups of transmembrane helices existed in the N- and C-termini and a highly heterogeneous hydrophilic region was located at the centre in most ClPIN and ClABCB proteins (Additional file 4: Figure S1). The PIN protein hydrophilic loop is partially modular for the trafficking behaviour and the intracellular trafficking is plastic depending on cell type and developmental stage [60]. The presence of the hydrophilic region in PIN and ABCB proteins from watermelon suggested that they had a similar trafficking behaviour to *Arabidopsis*. Phylogenetic and domain structural analyses showed that PIN and ABCB protein functions were conserved between watermelon and *Arabidopsis* [61].

### Tissue-specific expression analysis of *ClLAX*, *ClPIN* and *ClABCB* genes

Tissue-specific expression analysis of *ClLAX*, *ClPIN* and *ClABCB* genes indicated that the transcriptional level of these auxin transporter genes expressed in the roots, cotyledons, leaves, shoots and flowers varied greatly. *LAX*, *PIN* and *ABCB* genes have been found to be involved in plant growth and development previously [16, 43, 57]. The differential expression level of most of *ClLAX*, *ClPIN* and *ClABCB* genes in different tissues showed that they might be involved in the regulation of growth and development in watermelon. In spite of the conservation in protein structure, the *ClLAX* expressed among tissues/organs with different intensities. The high identity of *LAX* genes between watermelon and *Arabidopsis* at the protein level indicated that *ClLAX* genes might have conserved function as their *Arabidopsis* orthologue genes (Additional file 2: Table S2). In *Arabidopsis*, four *AUX/LAX* genes have complementary and non-redundant expression profiles in the roots and facilitate distinct developmental process: *AtAUX1* functions in root gravitropism [12] and root hair development [62]; *AtLAX2* functions in vascular development and cell division in the QC [16, 17]; *AtLAX3* and *AtAUX1* coordinately regulates apical hook development [63] and lateral root development [13]. The *ClLAX* genes might play similar or different roles during watermelon development because of their variety of expression patterns. *PIN* family genes have been previously elucidated to participate in growth and development in a variety of plant species [27]. *AtPIN1* is expressed during early embryonic development. Later, it expressed in the primary root and in the inflorescence stems [33]. Three *OsPIN5* homologous genes exist in rice genome. *OsPIN5a* and *OsPIN5c* weakly expressed in roots, highly expressed in leaves, shoot apex, and panicle. *OsPIN5b* expressed in young panicles and may be involved in inflorescence formation in rice [37]. *ZmPIN1b*, an orthologue of *AtPIN1*, is highly expressed during female inflorescence development in maize [34]. Our data showed that two *ClPIN* genes (*ClPIN3* and *ClPIN10*) were more highly expressed in the roots than in any other tissues, suggesting that they may function in root development. The subclass B of the ABC superfamily includes the majority of proteins that are able to bind and transport auxin in *Arabidopsis*. However, other members transport other substrates. The *AtABCB14* was first described as a malate transporter [49]. To date, there has been no functional characterization of the ABCBs in watermelon and the likely role of members in auxin transport. We sought to identify candidate *ClABCB*s with the function of auxin transport. Our phylogenetic analysis showed that the ClABCB11 and ClABCB14 cluster along with AtABCB19 and AtABCB1, respectively, both of which were known as IAA transporters. Further investigation, including cell-type specific expression pattern analysis of these family genes and expression patterns during different developmental processes, is required to reveal how these genes participated in the development regulation functions.

### Expression patterns analysis of *ClLAX*, *ClPIN* and *ClABCB* genes upon IAA treatment

To determine whether the auxin transporters were involved in auxin signal, we analysed the gene expression profiles of these genes at different times under IAA treatment. In *Arabidopsis*, *AtLAX1* and *AtLAX3* were highly induced by 2, 4-D in the roots [16]. The expression of *AtPIN6* is upregulated by auxin though repressive chromatin modification [64]. The expression level of *AtABCB4* is enhanced by 2, 4-D treatment [65] and *AtABCB1* is also up-regulated by exogenous auxin application [41]. *OsABCB14* was induced rapidly by exogenous auxin in rice. The expression of *OsPIN1a* showed a fivefold increase after IAA treatment [37]. In maize, most of the auxin transporter genes responded to auxin treatment in both shoots and roots [24]. *ClPIN5* and *ClPIN7*, two orthologue genes of *AtPIN1* in watermelon, were also drastically induced by IAA treatment. *ClABCB14* and *ClABCB11*, the orthologue of *AtABCB1*and *AtABCB19* in watermelon, respectively, were up-regulated after IAA treatment in the roots.

### *ClLAX*, *ClPIN* and *ClABCB* genes were related to salt, drought, cold and grafting response

As one of the most important phytohormones, auxin regulates plant growth and mediates various environmental stress responses by controlling several auxin-responsive genes. Recently, evidence has indicated that environmental stresses change auxin distribution and homeostasis mediated by auxin transporters [66, 67]. It has been reported that various abiotic signals can change auxin distribution by modulating the expression of auxin transporter genes [66]. In soybean, abiotic stress and hormonal treatments altered auxin accumulation and distribution in the roots. In addition, under these conditions, some *GmPIN* genes might contribute to auxin distribution and homeostasis [68]. In rice, overexpression of *OsPIN3t* improved drought tolerance and knockdown of *OsPIN3t* led to insensitive to drought stress [36]. Therefore, auxin transporters might mediate the crosstalk between auxin and abiotic stresses. The majority of the *ClLAX*, *ClPIN* and *ClABCB* genes were responsive to cold, drought and high salinity both in the shoot and root tissues. The expression profiling of *ClLAX*, *ClPIN* and *ClABCB* genes changed under abiotic stresses, which might accelerate or decelerate the transportation of endogenous auxin in watermelon seedlings. The responses of auxin transport genes to highly saline and drought stress and their different expression profiles indicated that the transcriptional expressions of these auxin transporter genes were regulated by the different physiological signals. Auxin redistribution and transport may be required for watermelon when it responded to abiotic stresses.

Low-temperature stress is a common adversity, which is often encountered in plant cultivation [69]. Many studies have indicated that a relationship between auxin and low temperature stress [70]. Cold stress changes the growth and development of plants closely related to the intracellular concentration gradient of auxin, which is regulated by asymmetric localisation and intracellular trafficking of auxin carriers. For example, the asymmetric redistribution and intracellular cycling of AtPIN3 protein were blocked by cold stress. Cold stress also inhibits the intracellular cycling of AtPIN2 [71]. During low temperature stress, the immobilisation of PINs represents a selective process to regulate the activity of specific proteins, which provides a mechanistic basis to explain the role of auxin in regulating the growth and development of plant under cold stress. Watermelon is an annual herb of the gourd family, originating from tropical Africa. Most varieties of watermelon are weak to cold hardiness and vulnerable to seasonal restrictions. The transcriptional level of most *ClLAX*, *ClPIN* and *ClABCB* genes also changed during the cold treatment, which suggested that these genes may function in the mechanism that helps watermelon tolerate cold stress.

Auxin plays a pivotal role in development, and the mode of auxin flow through a tissue determines the sites of vein formation [72]. Similarly, auxin promotes the formation of the xylem and phloem in callus [73]. In *Arabidopsis*, normal vein development depends on polar auxin transport and can be modified by auxin transport inhibitors or mutations of auxin transport genes [74]. The expression levels of most *ClLAX*, *ClPIN* and *ClABCB* genes also changed during grafting. This condition suggested that these genes might play a significant role in auxin transported to graft junction, thereby promoting wound healing and vascular formation.

Some members of auxin transporter family genes have been found to engage in the response to abiotic stresses (such as alkaline, drought, heavy metal, high salinity, nutritional deficiency and cold stress). In *Arabidopsis*, AtAUX1 played an important role in plant tolerance to oxidative stress caused by arsenite [75]. Shoot-supplied ammonium influenced root architecture by interfering with AUX1-dependent auxin transport [76]. Auxin homeostasis is changed in roots under cadmium stress via AUX1 proteins both in *Arabidopsis* and rice [22, 77]. Aluminium toxicity altered auxin distribution through AtPIN2 and AtAUX1 auxin transporter proteins [78]. *AtPIN2* helps roots adapt to alkaline stress by modulating root tip proton secretion [79]. The expression levels of *AtPIN3* and *AtPIN1* genes were reduced under oxidative stress caused by alloxan [80]. *AtABCB* genes responding to light, CO_2_, phytochromes and cryptochromes have rarely been reported [49, 81]. The expression of *SbLAX4* gene was dramatically reduced under salt and drought stresses [23]. These physiological and genetic evidence suggests that auxin transporters respond to abiotic stress. The expression profiling of *ClLAX*, *ClPIN* and *ClABCB* gene changes under abiotic stress may affect endogenous auxin redistribution and concentration. Auxin homeostasis is a crucial process for plant to adapt to changing environments. Further studies, including molecular biology and reverse genetics analysis of each watermelon auxin transporter, will extend our understanding of the regulation mechanisms between auxin transporters and abiotic stresses.

## Conclusions

In summary, we characterized the transcript pattern of *ClLAX*, *ClPIN* and *ClABCB* family genes in watermelon under exogenous IAA treatments or adversity stress. The distinct expressions of *ClLAX*, *ClPIN* and *ClABCB* genes indicated different regulatory action of these genes in watermelon tolerance to abiotic stresses.

## Additional files

Additional file 1: Table S1. List of qRT-PCR primers used in the present study. (DOCX 1082 kb)

Additional file 2: Table S2. Percent Identity Matrix of LAX family bweteen waternelon and *Arabidopsis*. (DOCX 648 kb)

Additional file 3: Table S3. qRT-PCR values of the *ClLAX*, *ClPIN* and *ClABCB* family genes in five tissues. (DOCX 1910 kb)

Additional file 4: Figure S1. Transmembrane helices of *ClLAX*, *ClPIN* and *ClABCB*. Protein transmembrane topology was analyzed using the TMHHM Server. (DOCX 1872 kb)
