# Supplementary material for: Genome-wide identification and expression analysis of ClLAX, ClPIN and ClABCB genes families in Citrullus lanatus under various abiotic stresses and grafting
Source: BMC Genet. 2017 Apr 7;18:33. doi: 10.1186/s12863-017-0500-z (PMC5384148; doi:10.1186/s12863-017-0500-z)
Supplement: Supplementary file 1 — List of qRT-PCR primers used in the present study. (DOCX 1082 kb) [file 12863_2017_500_MOESM1_ESM.docx]

**Additional file 1 Table S1** List of qRT-PCR primers used in the present study
