# Supplementary material for: Genome-wide identification and expression analysis of ClLAX, ClPIN and ClABCB genes families in Citrullus lanatus under various abiotic stresses and grafting
Source: BMC Genet. 2017 Apr 7;18:33. doi: 10.1186/s12863-017-0500-z (PMC5384148; doi:10.1186/s12863-017-0500-z)

**Additional file 2 Table S2** Percent Identity Matrix of LAX family bweteen waternelon and *Arabidopsis*


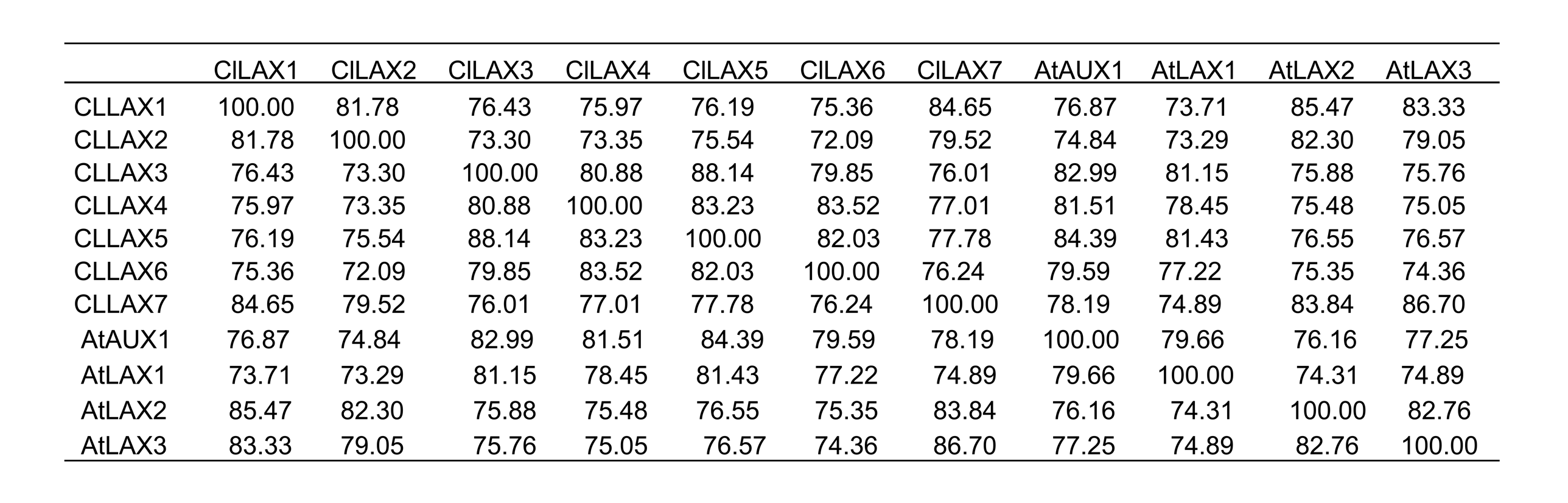

Supplement: Supplementary file 2 — Percent Identity Matrix of LAX family bweteen waternelon and Arabidopsis. (DOCX 648 kb) [file 12863_2017_500_MOESM2_ESM.docx]
