# Supplementary material for: Genome-wide identification and expression analysis of ClLAX, ClPIN and ClABCB genes families in Citrullus lanatus under various abiotic stresses and grafting
Source: BMC Genet. 2017 Apr 7;18:33. doi: 10.1186/s12863-017-0500-z (PMC5384148; doi:10.1186/s12863-017-0500-z)
Supplement: Supplementary file 3 — qRT-PCR values of the ClLAX, ClPIN and ClABCB family genes in five tissues. (DOCX 1910 kb) [file 12863_2017_500_MOESM3_ESM.docx]

**Additional file 3 Table S3** qRT-PCR values of the *ClLAX*, *ClPIN* and *ClABCB* family genes in five tissues
