# Supplementary material for: Genome-wide identification and expression analysis of ClLAX, ClPIN and ClABCB genes families in Citrullus lanatus under various abiotic stresses and grafting
Source: BMC Genet. 2017 Apr 7;18:33. doi: 10.1186/s12863-017-0500-z (PMC5384148; doi:10.1186/s12863-017-0500-z)
Supplement: Supplementary file 4 — Transmembrane helices of ClLAX, ClPIN and ClABCB. Protein transmembrane topology was analyzed using the TMHHM Server. (DOCX 1872 kb) [file 12863_2017_500_MOESM4_ESM.docx]

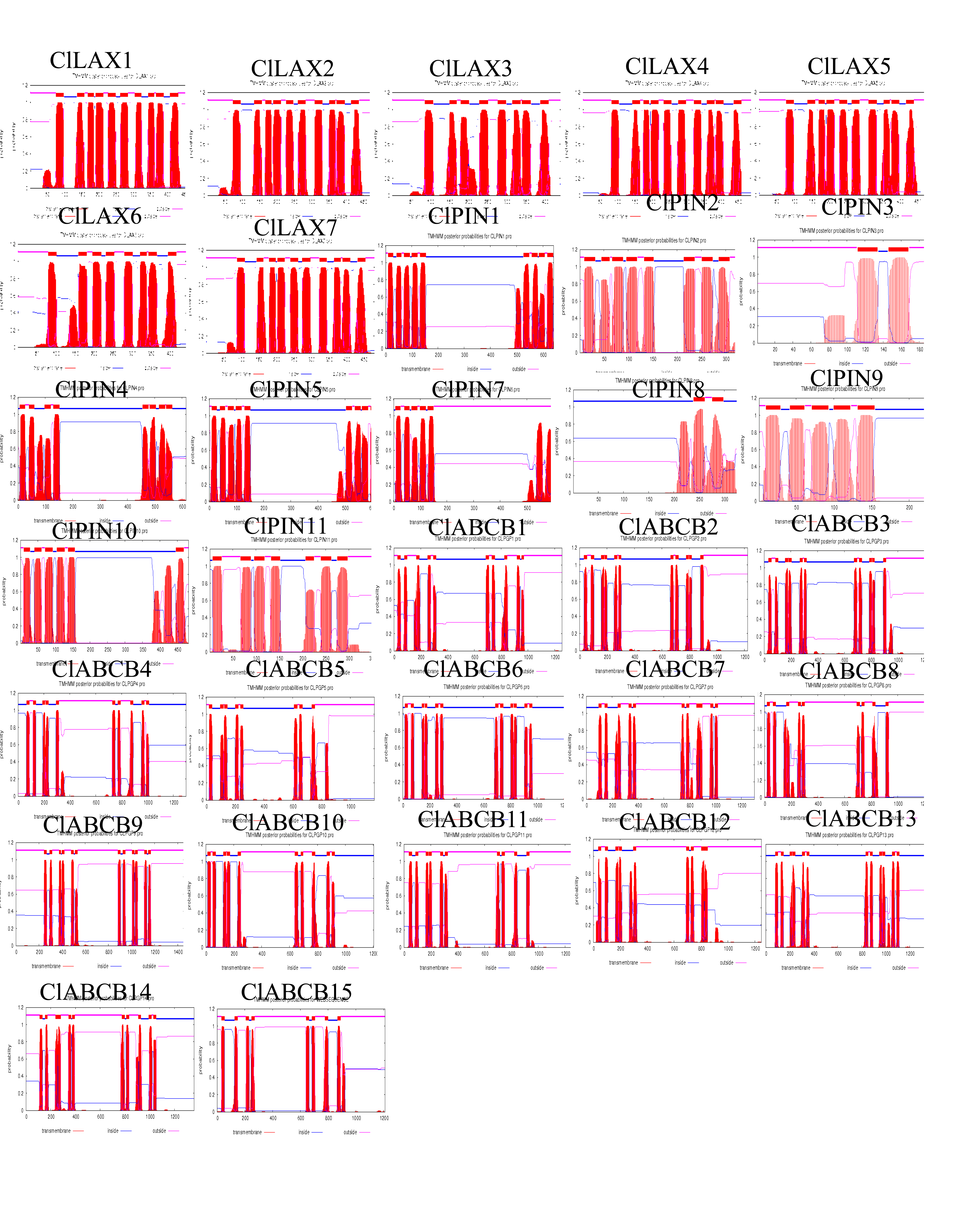


**Additional file 4 Figure S1** Transmembrane helices of *ClLAX*, *ClPIN* and *ClABCB*. Protein transmembrane topology was analyzed using the TMHHM Server.
